# The effects of genomic germline variant reclassification on clinical cancer care

**DOI:** 10.18632/oncotarget.26501

**Published:** 2019-01-11

**Authors:** Thomas P. Slavin, Sophia Manjarrez, Colin C. Pritchard, Stacy Gray, Jeffrey N. Weitzel

**Affiliations:** ^1^ Department of Medical Oncology and Therapeutics Research, Division of Clinical Cancer Genomics, City of Hope, Duarte, CA, USA; ^2^ Department of Population Sciences, City of Hope, Duarte, CA, USA; ^3^ Department of Laboratory Medicine, University of Washington, Seattle, WA, USA

**Keywords:** variant reclassification, BRCA1, BRCA2, genetic testing, hereditary cancer

## Abstract

The last two decades have provided an astounding amount of novel information about the human genome. Translating germline genomic data into clinically actionable findings is reliant on the annotation and laboratory classification of specific variants. Variant classification helps providers and patients determine if genomic findings can inform clinical management. In germline hereditary cancer predisposition testing, variants of uncertain significance (VUS) are routinely misunderstood. By definition, they cannot be classified by the testing laboratory as either problematic mutations or benign variants. Many VUS undergo category reclassifications over time (from months to years after initial classification) as more information is known about normal human genomic diversity, especially among underrepresented minority populations. When VUS are reclassified, it has been shown that they are often downgraded. Likewise, some variants originally thought to be actionable mutations are downgraded to VUS or benign variants. Rarely but importantly, VUS may be reclassified in a manner that increases their initial clinical significance. Here, we discuss the insights gained from the study of variant reclassification. We provide a case series to highlight the potential impact that variant reclassifications can have on individual and family cancer management, risk counseling, and screening.

## INTRODUCTION

**Figure 1:**
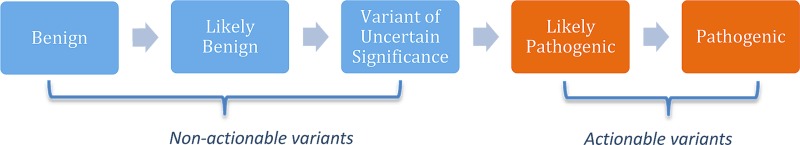
American college of medical genetics and genomics and the association for molecular pathology variant classification guidelines

Variants identified through genetic testing must be classified according to their clinical significance. Many different classification schemes exist. For germline variant annotation, variants are frequently classified using guidelines jointly established by the American College of Medical Genetics and Genomics (ACMG) and the Association for Molecular Pathology [1]. The ACMG guidelines classify variants in one of five tiers, from lowest to highest pathogenicity: benign, likely benign, variant of uncertain significance (VUS), likely pathogenic, and pathogenic. Variants in the pathogenic and likely pathogenic categories are treated as clinically actionable variants that could impact counseling and clinical care, whereas variants in the benign, likely benign, and VUS categories are treated as clinically non-actionable (Figure 1) [2, 3]. Variants can be reclassified over time, as new insights regarding their phenotypic effects are discovered. A change in variant reclassification can have profound implications for patient care.

### Variants are frequently reclassified and usually downgraded over time

We recently reported the rates of variant reclassification by ancestry in individuals undergoing hereditary cancer predisposition germline testing [2]. Non-benign variants in 42 actionable, commonly evaluated hereditary cancer predisposition genes, categorized per ACMG guidelines [1, 2], were identified in consenting participants referred for cancer genetic evaluation at two Southern California sites from September 1996 to December 2016. Over the course of the study, genetic testing for each site was conducted in multiple commercial laboratories. Variants were followed for reported reclassifications through February 2017. Overall, 1743 participants had 1816 variants analyzed. Of these, 294 individuals (16.9%) had 315 variants (17.3%) reclassified. Fifty-one variants were reclassified more than once. Variant reclassification occurred between 63 days and 20.2 years after initial classification, with a median of 3.55 years [2].

Our findings showed that for non-benign variants in cancer-related genes, the rate of reclassification varied by ancestry and in ways that differed between *BRCA*1/2 and other genes [2]. Rates of reclassification overall were highest in minorities. Similar to recent publications on variant reclassifications [4–6], we determined that the vast majority of unique variant reclassifications were downgrades (90.3%) with a much smaller fraction (9.7%) being upgraded. In particular, only 7.5% of variants reclassified from the VUS category were upgraded.

The disproportionate number of downgrades from the VUS category seen in our cohort, in addition to data from other recent publications [4, 6], shows the aggressive usage of the VUS category by laboratories during variant annotation. If the VUS category was used impartially, the number of upgraded and downgraded reclassifications from this category would be more evenly balanced. This overuse of VUS classification is becoming an even greater issue because VUS rates track with the number of genes tested [7] and the use of multigene panels has rapidly expanded in standard of care hereditary cancer predisposition testing [8].

Furthermore, genetics education regarding variant interpretation amongst providers is lacking [9]. Even though a VUS is recommended to be handled as a non-actionable finding [3], it has been shown that some providers inappropriately treat this category similarly to pathogenic variants, leading to mismanagement of patients, inappropriate cancer screening, and unnecessary surgical procedures (e.g., bilateral prophylactic mastectomy and bilateral oophorectomies) [10, 11]. Furthermore, patients often misinterpret the meaning of a VUS and may use the result erroneously to inform medical decision-making and cancer screening behavior [12]. Ideally, as variant annotation improves, the VUS category will shrink in comparison to the other more definitive categories.

### The clinical impact of variant reclassifications

We now provide a complimentary case series (Table 1) to highlight the potential clinical impact of variant reclassification on patients and their families from the original cohort described above [2]. To emphasize the impact variant reclassification may have on families, this new analysis included variants seen multiple times in the same family. Of the variants that underwent any reclassification, only 25/322 (7.8%) in 25 patients resulted in a change in actionability. Of all variants that resulted in a change in actionability, 16 (64%) were actionable upgrades (benign, likely benign, or VUS reclassified to likely pathogenic or pathogenic categories), and 9 (36%) were actionable downgrades (likely pathogenic or pathogenic reclassified to benign, likely benign, or VUS categories) (Figure 1, Table 1). These reclassifications involved 9 genes (*BRCA1*, *BRCA2*, *BRIP1*, *MET*, *MLH1*, *MSH2*, *NBN*, *PTEN*, and *SDHB*). Overall, 12 cases (48%) involved *BRCA1* and *BRCA2*. The majority of participants (14; 56%) reported non-Hispanic European ancestry, and the remaining 11 (44%) were from a minority group.

**Table 1:** Case series of actionable variant reclassifications

Pts	Gene	Variant	Type	Sex	Age at End of Study	Self Reported Race	Maternal - Paternal Ancestry	Affected status (age at diagnosis)	Summary of Reclassifi-cation Events	Adult Living FDRs		
										Total	Women	Men
**Upgrades**												
1	BRCA1	p.Arg1495Lys	Missense	F	41	Other	Salvadorean - Salvadorean	BR (39)	VUS-->LP-->P	5	3	2
2	BRCA1	p. Arg1495Met	Missense	F	D @ 51	White	Italian - Italian	Ov (46)	VUS-->P	2	1	1
3	BRCA1	p.Thr1691Lys	Missense	F	59	Asian	Chinese/Indonesian - Chinese/Indonesian	DCIS (47)	VUS--LP	11	5	6
4	BRCA1	p.Gly1706Glu	Missense	F	59	White	Armenian - German	Br (31) Br (42) Br (51)	VUS--LP	4	3	1
5	BRCA2	p.Trp2626Cys	Missense	F	45	White	English/Scottish/Swedish - Norwegian	Br (40)	VUS-->LP	4	1	3
6	BRCA2	p.Gly2793Arg	Missense	F	75	Other	Mexican - Mexican	Br (43) Br (60)	VUS-->LP--P	19	8	11
7	BRCA2	c.8754+4A>G	Splice site/intronic	F	D @ 53	White	Italian - Italian	Br (37) Ov (52)	VUS-->LP--P	3	1	2
8	BRCA2	p.Arg3052Trp	Missense	F	50	Other	Mexican - Guatemalan/Mexican	Br (34) Br (45)	VUS-->P	3	2	1
9	BRCA2	p.Asp3095Glu	Missense	F	46	White	Unknown - Unknown	Br (37)	VUS-->P	4	2	2
10	BRIP1	c.3196delT	Nonsense/frameshift	F	42	African American	African American - African American	Br (41)	VUS-->LP	3	1	2
11	MLH1	p.Thr117Met	Missense	F	58	White	Irish - Irish	Br (39) CRC (40) UT (40)	VUS-->P	4	1	3
12	MSH2	p.Ser554Arg	Missense	F	51	American Indian/ Alaska Native	Native American - Native American	Sebaceous skin neoplasia (39)	VUS-->LP	6	2	4
13	MSH2	p.Asn596del	inframe indel	M	D @ 79	White	Scottish/Irish - German	CRC (57) Pan (57) CRC (69) T Cell Lymphoma (74)	VUS-->P	3	3	0
14	MSH2	p.Asn596del	inframe indel	F	59	White	Scottish/Irish - German	SqCC (38) Ut (44) Colon polyps (40)	VUS-->P	3	2	1
15	MSH2	p.Ala636Pro	Missense	F	69	White	Russian - Russian	CRC (46) Ut (53) Sebaceous skin neolasia (64) Sarcoma (70)	VUS-->LP	5	2	3
16	SDHB	p.Ile127Ser	Missense	M	57	White	Irish/Scottish - Irish/Scottish	Paraganglioma (25) Pheochromocytoma (26)	VUS-->P	6	4	2
**Downgrades**												
1	BRCA1	c.4096+1G>A	Splice site/intronic	F	68	Other	Syrian - English	Br (46)	VUS-->LP-->P-->VUS	7	6	1
2	BRCA1	c.4096+1G>A	Splice site/intronic	F	64	Other	Syrian - English	Br (45)	LP-->P-->VUS	6	4	2
3	BRCA1	c.4096+1G>A	Splice site/intronic	F	41	Other	Syrian - English	Unaffected	LP-->P-->VUS	4	3	1
4	MET	c.1200+2T>C	Splice site/intronic	F	71	White	Irish/Scandinavian/Spanish - European	Br (68) Colon Polyps (68)	LP-->VUS	9	4	5
5	MLH1	dup exons 16-19	Exonal duplication(s)	F	54	White	Armenian/Syrian - Armenian	Unaffected	LP*-->VUS	8	7	1
6	MSH2	p.Met1Leu	Missense	F	63	White	Unknown - Unknown (West European)	Unaffected	LP-->P-->VUS	6	4	2
7	MSH2	dup exons 1-4	Exonal duplication(s)	F	81	Asian	Taiwanese - Taiwanese	CRC (66) Ut (71) Lung (76)	LP-->VUS	11	6	5
8	NBN	p.Arg215Trp	Missense	F	48	Other	Mexican - Mexican	Br (45)	P-->VUS	11	5	6
9	PTEN	p.Ala79Thr	Missense	F	55	White	Italian - Croatian/Serbian	Unaffected	P-->VUS	3	1	2
**Total relatives**										**150**	**81**	**69**

Abbreviations: Participants (pts); deceased (D); breast (Br), colorectal cancer (CRC), uterine (Ut), ovarian (Ov), ductal carcinoma in situ (DCIS), pancreatic (Pan), squamous cell cancer of the skin (SqCC); reclassifications: pathogenic (P), likely pathogenic (LP), variant of uncertain significance (VUS); first-degree relative (FDR). It should be noted that some individuals had multiple rounds of reclassification, one that started and ended in the VUS category.

If pathogenic or likely pathogenic variants (mutations herein) are initially classified as VUS, likely benign, or benign variants, the resulting delay in the identification of a clinically actionable variant can have profound effects on patient care. These delays can adversely affect decision making related to prophylactic and treatment-related surgeries, cancer screening and prevention in unaffected individuals, and the use of genetically targeted therapy in cancer patients. For instance, there is a clinical urgency for patients with newly diagnosed breast cancer to understand their genetic risk to help inform breast cancer surgical decision-making [13]. In our sample, of the *BRCA1* or *BRCA2* variants that were upgraded to actionable, all were in women (Table 1). The initial classification of *BRCA1* or *BRCA2* variants as non-actionable may have delayed consideration of risk reducing bilateral mastectomy (RRM) and risk reducing salpingo-oophorectomy (RRSO) to reduce future breast and ovarian cancer risk [3]. Furthermore, poly-ADP ribose polymerase (PARP) inhibitors as a chemotherapeutic option may not have been considered for cancer treatment [14, 15]. Similarly, a delay in the classification of the *BRIP1* mutation as actionable may have delayed RRSO to mitigate the elevated risk of ovarian cancer [16]. In patients with *MLH1* and *MSH2* variants, Lynch syndrome-associated cancer screening, including colonoscopies every one to two years, consideration of total abdominal hysterectomy (TAH) with RRSO in females, upper endoscopies, and renal and pancreatic cancer screening [17], may have been delayed. The carrier of the *SDHB* pathogenic variant may not have known until too late that, even if their current tumors were treatable, they were at high risk for future paragangliomas, pheochromocytomas, and renal cancer, delaying annual screening for these cancers [18].

The downgrade of variants may have also led to substantial changes in management. Specifically, upon receiving a pathogenic or likely pathogenic result, patients may be counseled to engage in screening or preventative care, or to undergo prophylactic surgery, and that such advice, in retrospect, may be clinically inappropriate. For example from Table 1, prior to reclassification to VUS, carriers of downgraded *BRCA1* variants would have been recommended to undergo a RRSO and initiate high risk breast cancer screening or complete RRM. High risk breast cancer screening would also have been recommended to the individual with the *NBN* variant prior to its downgrade [3]. Individuals with the downgraded Lynch syndrome variants (*MLH1*, *MSH2*) would have been recommended to undergo Lynch syndrome-associated cancer screening (noted above) instead of screening based on their family history of cancer alone. The individual carrying the downgraded *MET* variant would have been recommended to undergo close kidney screening by imaging [19]. The individual with the *PTEN* variant would have been recommended to follow Cowden syndrome screening guidelines, which includes high-risk breast cancer screening or consideration of RRM, enhanced colon cancer screening, TAH, and annual thyroid and renal cancer screening [3]. Importantly, four of nine (44.4%) patients with downgraded variants were unaffected by cancer. Unlike carriers already being treated for cancer, these individuals would receive cancer risk and screening recommendations highly dependent on their variant classification.

Additionally, even when considering just the first-degree relatives, the potential impact of the changes in actionability of the reclassified variants is impressive. The reclassifications we followed may have affected the genetic cancer risk assessment/counseling for 150 adult male and female first-degree relatives of the 25 carriers. If half of these individuals are assumed to carry the reclassified variant due to the autosomal dominant transmission of the genes listed in Table 1, 75 individuals would be at risk of overtreatment or missed opportunities for cancer screening or risk reducing procedures. Beyond the health implications that genetic testing has for the tested individual and his or her at-risk relatives, variant classification can also influence reproductive decision making. For example, tested individuals and at-risk relatives who are conceiving children may make important decisions about child bearing and preimplantation genetic testing based on the results of genetic testing. Some individuals may choose not to conceive children based solely on knowing there is a cancer-causing mutation in the family. Alternatively, some prospective parents who have mutations in genes like *BRCA1*, *BRCA2*, *MLH1*, etc., consider preimplantation genetic diagnosis to reduce the risk of genetic transmission to offspring [3, 17]. Furthermore, known carriers of pathogenic or likely pathogenic variants in genes for Fanconi anemia (in our cases *BRCA1*, *BRCA2*, *BRIP1*) [3] and constitutional mismatch repair deficiency syndrome (Lynch syndrome genes) [17] would be recommended to undergo prenatal counseling and may have their partners tested to understand and/or reduce the risk of these types of severe autosomal recessive childhood-onset conditions. If variants are initially misclassified, prospective parents may either unnecessarily opt for, or fail to engage in, medically appropriate and preference sensitive reproductive decision making.

Because our sample set included multiple family members, the *MSH2* p.Asn596del and *BRCA1* c.4096+1G > A variants were each detected in more than one relative; therefore, the true number of impacted first-degree relatives may be slightly lower than reported. However, this does not substantially diminish the potential effect of variant reclassifications on families. Of note, the classification of the *BRCA1* c.4096+1 variant was particularly volatile, starting as a VUS in the first person tested in the family in our cohort (downgrade participant 1, Table 1). The variant was subsequently reclassified three times, first upgraded to an actionable variant prior to being ultimately downgraded to a VUS prior to study closure. Two other family members (downgrade participants 2 and 3, Table 1) had the same variant initially classified as likely pathogenic before downgrade.

In addition to differences in variant calling over time, there may be differences depending on the testing laboratory. According to the ClinVar database [20], classification of the *BRCA1* c.4096+1 variant is discordant amongst many large national commercial hereditary cancer predisposition laboratories and expert variant curation groups, with some groups calling it a VUS and others a likely pathogenic or pathogenic variant. Therefore, individuals being tested for this variant in the same family may be given inconsistent information regarding the actionability of the variant if tested through different commercial laboratories. This demonstrates how inconsistent variant calling can create potential confusion within a family regarding the clinical actionability of a shared variant, highlighting yet another challenge of variant interpretation [21, 22] and the need to harmonize variant calling amongst major commercial germline testing laboratories.

Overall, the above implications of variant reclassification in families are only amplified when one considers cascade testing and decision making beyond first-degree relatives.

### Passive *versus* active reclassification methods

Our research and clinical experience has also revealed that the reclassification of variants is highly dependent on the testing laboratory. It is imperative that providers who order genetic testing understand how each testing laboratory approaches reclassification. Some commercial genetic testing laboratories employ an active variant reclassification process, conducting periodic computational reviews of all variants in their databases to continuously reclassify variants. If variants are deemed in need of reclassification, all providers on record for the patients affected by the reclassification are notified. Other laboratories use passive reclassification processes, in which the providers must supply new information to help determine whether a specific variant is benign or pathogenic. This information could take the form of literature, variant tracking, and/or other family studies or patient phenotypic data, such as commercial splice site analysis using RNA. Many laboratories use a mixture of these two techniques for variant reclassification. We strongly recommend that the genetics community and clinically active testing laboratories work to standardize variant reclassification processes to enable consistent systematic active variant reclassifications and provider/patient notifications.

## CONCLUSIONS

Variant reclassification is commonplace in hereditary cancer predisposition testing. The majority of VUS category variants will be downgraded over time and should not be treated as positive mutations in any event. In some cases, counseling may be nuanced if there is a strong clinical phenotype and/or suggestive ancillary data, but the variant does not quite meet criterion for likely pathogenic or pathogenic classification. In some of those cases, clinical management is driven by the clinical phenotype rather than the variant itself. Few reclassifications entail changes in actionability; however, these reclassifications may substantially affect patients' options for cancer prevention, screening, targeted cancer therapy, surgical decisions and reproductive decision making. Variant reclassifications also have profound implications for the family members of tested individuals through cascade testing and subsequent medical recommendations and health-related decision making. Providers who order hereditary cancer genetic testing should consider implementing standard practices to appropriately identify and manage reclassified variants (Table 2). Continued research to improve the accuracy of initial variant classification is needed. In addition, examining the effects of patient and provider perceptions and the ultimate outcomes of variant reclassification on clinical care for patients and their families will be important. Data on variant reclassification is most robust in hereditary cancer genetics, however, it is likely that reclassifications will affect all aspects of diagnostic and/or prognostic genetic testing for both germline and somatic disorders and conditions.

**Table 2:** Variant reclassification considerations/recommendations

Variant reclassification considerations/recommendations for those ordering genetic testing
1. Consider using laboratories that have an active variant reclassification follow-up program.
2. Develop a standard operating procedure to notify patients and update the medical record for all reclassified variants.
3. Let patients know to update their contact information with you if address changes occur.
4. Suggest all patients with variants follow-up every few (~3-5) years for new information regarding their variants or consideration for update testing.
5. If the patient is being seen as a second opinion, consider taking steps to become listed as a managing provider for their testing in case of any reclassifications.
6. Maintain an element of skepticism for variants that do, or do not, fit a particular phenotype.
